# Influence of patient characteristics and oral lead-in on long-acting cabotegravir and rilpivirine pharmacokinetics and outcomes in people with HIV: a real-world study

**DOI:** 10.1128/aac.00145-25

**Published:** 2025-06-05

**Authors:** Marta Fernández-González, Guillermo Telenti, Christian Ledesma, María Losada-Echeberría, Enrique Barrajón-Catalán, Javier García-Abellán, Leandro López, Melissa Bello-Perez, Sergio Padilla, Mar Masiá, Félix Gutiérrez

**Affiliations:** 1 Infectious Diseases Unit, Hospital General Universitario de Elche 16686, Elche, Spain; 2 CIBERINFEC, Instituto de Salud Carlos III 38176, Madrid, Spain; 3 Fundación para el Fomento de la Investigación Sanitaria y Biomédica de la Comunitat Valenciana (FISABIO), Valencia, Spain; 4 Institute for Research, Development and Innovation in Health Biotechnology of Elche (IDiBE), Universidad Miguel Hernández, Elche, Spain; 5 Department of Clinical Medicine, Universidad Miguel Hernández, Alicante, Spain; IrsiCaixa Institut de Recerca de la Sida, Barcelona, Spain

**Keywords:** long-acting cabotegravir and rilpivirine, oral lead-in, pharmacokinetics, drug concentration monitoring, variability, virological failure

## Abstract

Factors influencing suboptimal pharmacokinetics (PK) of long-acting (LA) cabotegravir (CAB) and rilpivirine (RPV) in people with HIV (PWH) remain poorly understood. Lower plasma concentrations in underrepresented populations in clinical trials and direct initiation without an oral lead-in (OLI) require further investigation. This study examined CAB and RPV plasma levels in a real-world cohort, focusing on OLI’s role. This prospective cohort study followed PWH transitioning from oral antiretroviral therapy to CAB + RPV injections. Participants were sequentially assigned to start with injections (SWI) or with OLI. Plasma CAB and RPV levels were measured before each injection over 7 months, with virological monitoring up to 11 months. Mixed-effects models were used to identify the predictors of lower CAB and RPV levels, adjusting for OLI use, age, sex, body mass index (BMI), and smoking. Among 172 participants (median age: 48 years, 12.8% female, 14.7% BMI ≥30 kg/m², 45.5% smokers), 81 (47.1%) initiated therapy via SWI, and 91 (52.9%) via OLI. CAB concentrations were lower in participants with higher BMI (*P* = 0.019), male sex (*P* = 0.021), and smoking (*P* = 0.057). RPV levels were reduced in smokers (*P* = 0.011) and younger participants (*P* = 0.064). PK profiles and virological outcomes were similar between SWI and OLI groups, with trough concentrations above inhibitory thresholds. Rates of virological failure (1 SWI vs. 2 OLI; *P* = 0.999) and low-level viremia (18.5% vs. 17.6%, *P* = 0.999) were comparable. Sex, BMI, and smoking influenced CAB and RPV levels, whereas OLI use had no significant impact on PK or virological outcomes, supporting flexible CAB +RPV LA initiation strategies.

## INTRODUCTION

Long-acting injectable (LA) antiretroviral therapy (ART) has transformed HIV care by offering people with HIV (PWH) an alternative to daily oral regimens, potentially improving adherence, convenience, and quality of life. Among LA therapies, the combination of cabotegravir (CAB), an integrase strand transfer inhibitor (INSTI), and rilpivirine (RPV), a non-nucleoside reverse transcriptase inhibitor (NNRTI), administered bimonthly, has demonstrated high efficacy in maintaining viral suppression with a favorable safety profile (1–4).

Initially, CAB + RPV LA therapy included a 4-week oral lead-in (OLI) phase, wherein patients receive oral CAB and RPV before transitioning to injections. However, recent data from the FLAIR and SOLAR trials (4, 5) suggest that direct initiation with injections (start with injections [SWI]) yields comparable virological outcomes to OLI. Although omitting OLI simplifies treatment and reduces barriers to care, questions remain about its effect on pharmacokinetics (PK) and treatment outcomes (6).

Beyond initiation strategies, emerging evidence indicates that patient-specific factors—such as body mass index (BMI), smoking status, and sex—may play a significant role in PK variability (6–10). For example, higher BMI and smoking have been linked to lower CAB and RPV plasma concentrations (6, 8–10), whereas sex differences in CAB exposure have been observed, with women showing lower trough concentrations (Ctrough) during initiation but higher levels during maintenance (7–9). These factors may impact drug distribution and metabolism, potentially influencing virological outcomes.

Given these complexities, understanding how initiation strategies and individual patient characteristics affect CAB + RPV LA therapy is critical for optimizing treatment. This study aims to evaluate the relative impact of the OLI phase and patient-specific factors on PK and virological outcomes in a real-world cohort transitioning from oral ART to CAB + RPV LA therapy. By identifying key determinants of drug exposure and treatment success, this research seeks to inform personalized, patient-centered approaches to LA therapy initiation.

## MATERIALS AND METHODS

### Study design and participants

This prospective cohort study, conducted at Hospital General Universitario de Elche in Spain between January and December 2023, evaluated the PK and virological outcomes of LA CAB + RPV in PWH transitioning from oral ART to bimonthly injectable regimens, including a 1-month LA loading dose. Participants were sequentially assigned to the SWI group (January–April 2023) or the OLI group (May–December 2023) based on the time period of their enrollment. Eligible participants were adults (≥18 years) with documented HIV-1 infection, stable ART for ≥6 months, and HIV-1 viral load (VL) <50 copies/mL for ≥3 months prior to enrollment. Exclusion criteria included chronic hepatitis B virus (HBV) infection and a history of virological failure on NNRTIs or INSTIs.

Electronic medical and pharmaceutical records were reviewed to identify potential drug-drug interactions (DDIs). Comedications with contraindications or potential DDIs with long-acting CAB + RPV were assessed using the University of Liverpool HIV Drug Interaction Database.

The SWI group transitioned directly to LA CAB + RPV injections, whereas the OLI group received daily oral CAB (30 mg) and RPV (25 mg) for 4 weeks before starting injections. For both groups, LA therapy was initiated with a loading dose of CAB (600 mg) and RPV (900 mg) intramuscularly, followed by maintenance doses of 600 mg/900 mg every 2 months, beginning 1 month after the loading dose. All participants in the study received care at the Infectious Diseases Unit of the Hospital General Universitario de Elche, where a team of three expert nurses and infectious disease specialists managed their treatment. Injections were administered exclusively at the dorsogluteal (DG) site by experienced nurses using the Z-track method. For patients with a BMI ≥ 30 kg/m², longer needle lengths (2 inches) were used to ensure proper drug delivery.

### Study procedures and laboratory assessments

Blood samples were collected from participants before each injection visit to measure plasma CAB and RPV Ctrough and HIV-1 VL. HIV-1 VL was quantified using the COBAS HIV-1 Test on the Cobas 6800 System (Roche Diagnostics SL, Barcelona, Spain). This assay quantifies VL in the range of 20–10,000,000 copies/mL and reports qualitative results for VLs < 20 copies/mL. Virological outcomes assessed included virological failure, defined as either two consecutive VLs ≥ 200 copies/mL or a single VL ≥1,000 copies/mL, and low-level HIV-1 viremia, defined as any VL ≥50 copies/mL.

After thermal inactivation (1 h, 56°C) of plasma samples, CAB and RPV trough concentrations were measured using a validated protocol (11), which involved protein precipitation and liquid chromatography coupled with triple quadrupole mass spectrometry (LC–QqQ-MS), following our previously established methodology (12). The lower limits of quantification (LLOQ) were 1.40 ng/mL for CAB and 1.78 ng/mL for RPV, as determined by the calibration curves specific to the assay used. Pharmacokinetic samples were collected over a 7-month period from the initiation of LA therapy. Virological follow-up continued for up to 11 months to assess virological failure and viral suppression.

### Statistical analysis

Baseline characteristics were compared between groups using Fisher or chi-square (χ²) tests for categorical variables and independent *t*-tests or Mann–Whitney U tests for continuous variables, depending on the data distribution. Linear mixed-effects models were employed to evaluate the impact of initiation strategy and patient-specific factors on pharmacokinetic outcomes, focusing on CAB and RPV trough levels winsorized at the 95th percentile to minimize the influence of outliers. The models adjusted for potential confounders, including age, sex, BMI, and smoking status. Random intercepts were included to account for between-subjects variability. All statistical analyses were performed using R software (version 4.0.3; R-Core Team 2020, R-4.1.2.1), with a two-sided *P*-value of <0.05 considered statistically significant.

## RESULTS

### Study population and baseline characteristics

The study included 172 participants, contributing a total of 698 plasma samples. The median age was 48 years (IQR: 38–56), with 12.8% female, 14.7% with BMI ≥30 kg/m², 45.5% smokers, and 77.3% of Spanish origin. The median duration of HIV diagnosis was 11.5 years (IQR: 4.9–22.9), the median VL at diagnosis was 4.78 log₁₀ copies/mL (IQR: 4.4–5.2), and the median nadir CD4 count was 270 cells/µL (IQR: 147–465). All participants were on stable ART, with 51.1% on an INSTI-based two-drug regimen. Of the cohort, 81 participants (47.1%) transitioned directly to LA injectable therapy (SWI), whereas 91 (52.9%) underwent a 4-week OLI phase before injections. Baseline characteristics, including age, sex, BMI, CD4 count, smoking status, and prior episodes of non-sustained viral suppression, were comparable between the SWI and OLI groups (Table 1).

**TABLE 1:** Baseline characteristics of participants transitioning to long-acting cabotegravir and rilpivirine injections according to initiation strategy^*a*^

Characteristic	All participants *N* = 172^*d*^	Oral lead-in group *N* = 91	Start with injection group *N* = 81	*P* value^*e*^
Age, median (IQR), years	48 (38–56)	47 (38–56)	50 (37–56)	0.514
Sex at birth, Female, n (%)	22 (12.8)	12 (13.2)	10 (12.3)	0.999
Race/Ethnicity, n (%)				0.360
White non-Hispanic	142 (82.6)	78 (85.7)	64 (79.0)	
Black	1 (0.6)	0 (0)	1 (1.2)	
Other	29 (16.9)	13 (14.3)	16 (19.8)	
Country of origin, n (%)				0.136
Spain	133 (77.3)	76 (83.5)	57 (70.4)	
Other West European countries	3 (1.7)	1 (1.1)	2 (2.4)	
Eastern European countries	4 (2.3)	1 (1.1)	3 (3.7)	
Latin American countries	29 (16.9)	13 (14.3)	16 (19.8)	
Other	3 (1.7)	0 (0)	3 (3.7)	
HIV transmission route**,** n (%)				0.439
Men having sex with men	101 (58.7)	54 (59.3)	47 (58.0)	
Heterosexual	31 (18.0)	17 (18.7)	14 (17.3)	
Injection drug use	19 (11.1)	12 (13.2)	7 (8.6)	
Other/Unknown	21 (12.2)	8 (8.8)	13 (16.1)	
Smoker, n/N (%)	76/167 (45.5)	47/91 (51.6)	29/76 (38.2)	0.112
BMI at transition, median (IQR), Kg/m^2^	24.8 (22.6–27.5)	24.8 (22.6–28.0)	24.9 (22.6–27.5)	0.818
BMI ≥ 30 Kg/m2 at transition, n/N (%)	25/170 (14.7)	15/91 (16.5)	10/79 (12.7)	0.627
HIV-1 Non-B subtypes, n/N (%)	17/92 (18.5)	13/53 (24.5)	4/39 (10.3)	0.106
Type B	75/92 (81.5)	40/53 (75.5)	35/39 (89.7)	
HIV-1 viral load at diagnosis (pre-ART), median (IQR), log_10_ copies/mL	4.78 (4.36–5.22)	4.70 (4.29–5.20)	4.82 (4.48–5.24)	0.437
≤4.9 log_10_ copies/mL, n/N (%)	68/124 (54.8)	40/69 (58.0)	28/55 (50.9)	0.546
>4.9 log_10_ copies/mL, n/N (%)	56/124 (45.2)	29/69 (42.0)	27/55 (49.1)	
Nadir CD4 count, median (IQR), cells/µL	270 (147–465)	278 (136–493)	262 (161–430)	0.667
Nadir CD4 <200 cells/µL**,** n/N (%)	56/169 (33.1)	30/89 (33.7)	26/80 (32.5)	0.998
Pre-transition ART regimen, n (%)				0.212
INSTI + 2 NRTI	36 (20.9)	20 (22.0)	16 (19.8)	
NNRTI + 2 NRTI	27 (15.7)	11 (12.1)	16 (19.8)	
PI + 2 NRTI	11 (6.4)	7 (7.7)	4 (4.9)	
DTG + 3TC	53 (30.8)	27 (29.7)	26 (32.1)	
DTG + RPV	35 (20.3)	17 (18.6)	18 (22.2)	
Two-drug non-INSTI regimen	9 (5.2)	8 (8.8)	1 (1.2)	
Other	1 (0.6)	1 (1.1)	0 (0)	
Time since HIV diagnosis, median (IQR), years	11.5 (4.9–22.9)	11.2 (4.9–22.4)	11.6 (4.9–22.9)	0.999
Time with HIV-1 viral load <50 copies/mL, median (IQR), years	5.1 (1.8–13.5)	6.3 (1.9–13.1)	4.6 (1.6–13.7)	0.700
Episodes of detectable viremia in the year pre-transition, n (%)				
At least one viral load ≥20 copies/mL	61 (35.5)	33 (36.3)	28 (34.6)	0.942
At least one viral load ≥50 copies/mL	24 (14.0)	15 (16.5)	9 (11.1)	0.427
At least one viral load ≥200 copies/mL	6 (3.5)	5 (5.5)	1 (1.2)	0.215
HIV-1 viral load at transition, n (%)				
Target not detected	115 (66.9)	61 (67.0)	54 (66.7)	0.999
<20 copies/mL	32 (18.6)	16 (17.6)	16 (19.7)	0.866
20–49 copies/mL	18 (10.4)	10 (11.0)	8 (9.9)	0.999
50–99 copies/mL	7 (4.1)	4 (4.4)	3 (3.7)	0.999
CD4 cell count at transition, median (IQR), cells/µL	702 (497–940)	700 (552–938)	717 (463–942)	0.275
CD4/CD8 ratio at transition, median (IQR)	0.92 (0.68–1.28)	0.93 (0.70–1.35)	0.91 (0.66–1.20)	0.375
RPV-associated mutations in historical medical records, n/N (%)	8^*d*^/107 (7.4)	3^*b*^/60 (5.0)	5^*c*^/48 (10.4)	0.462

^*a*^ ART, antiretroviral therapy; BMI, body mass index (calculated as weight in kilograms divided by height in meters squared); DTG, dolutegravir; IQR, interquartile range; INSTI, integrase strand transfer inhibitor; 3TC, lamivudine; n/N (%), denotes number of patients with the characteristic (n) out of the total number of patients with data available (N); NRTI, nucleoside reverse transcriptase inhibitor; NNRTI, non-nucleoside reverse transcriptase inhibitor; PI, protease inhibitor; RPV, rilpivirine.

^*b*^ E138A, V106I + E138A and E138A + V179D + P225H.

^*c*^ E138A (3 cases), V106I + E138A and E138G + V179E.

^*d*^ All of them conferring low-level resistance to RPV according to the Stanford University HIV Drug Resistance Database (https://hivdb.stanford.edu/).

^*e*^ Empty cells denotes that some *P* values are for polytomic characteristic (e.g. "HIV transmission route").

### Virological outcomes

At the initiation of LA therapy, viral suppression rates were similar between the groups. The proportion of participants with VL <50 copies/mL was 96.3% in the SWI group and 95.6% in the OLI group (*P* = 0.999).

Virological outcomes during the study period according to the initiation strategy are shown in Fig. S1. During the 11-month follow-up period, 82.0% of the participants maintained all VLs < 50 copies/mL. Median (IQ) number of VL ≥ 50 copies/mL per participant with low-level HIV-1 viremia was 1 (1–2). Virological failure occurred in three participants (1 SWI group, 2 OLI; *P* = 0.999), detailed in Table S1. Any VL ≥ 50 copies/mL was reported in 18.5% of SWI and 17.6% of OLI participants (*P* = 0.999). Fifteen participants (8.7%) discontinued CAB + RPV LA therapy during follow-up.

### Pharmacokinetic parameters

Pharmacokinetic analysis included 309 plasma samples from the 81 SWI group participants and 389 from the 91 OLI group participants, with 91 samples collected during the OLI phase. The distribution of all CAB and RPV trough concentrations determined in study participants over the 7-month study period is shown in Fig. S2.

**Fig 1:**
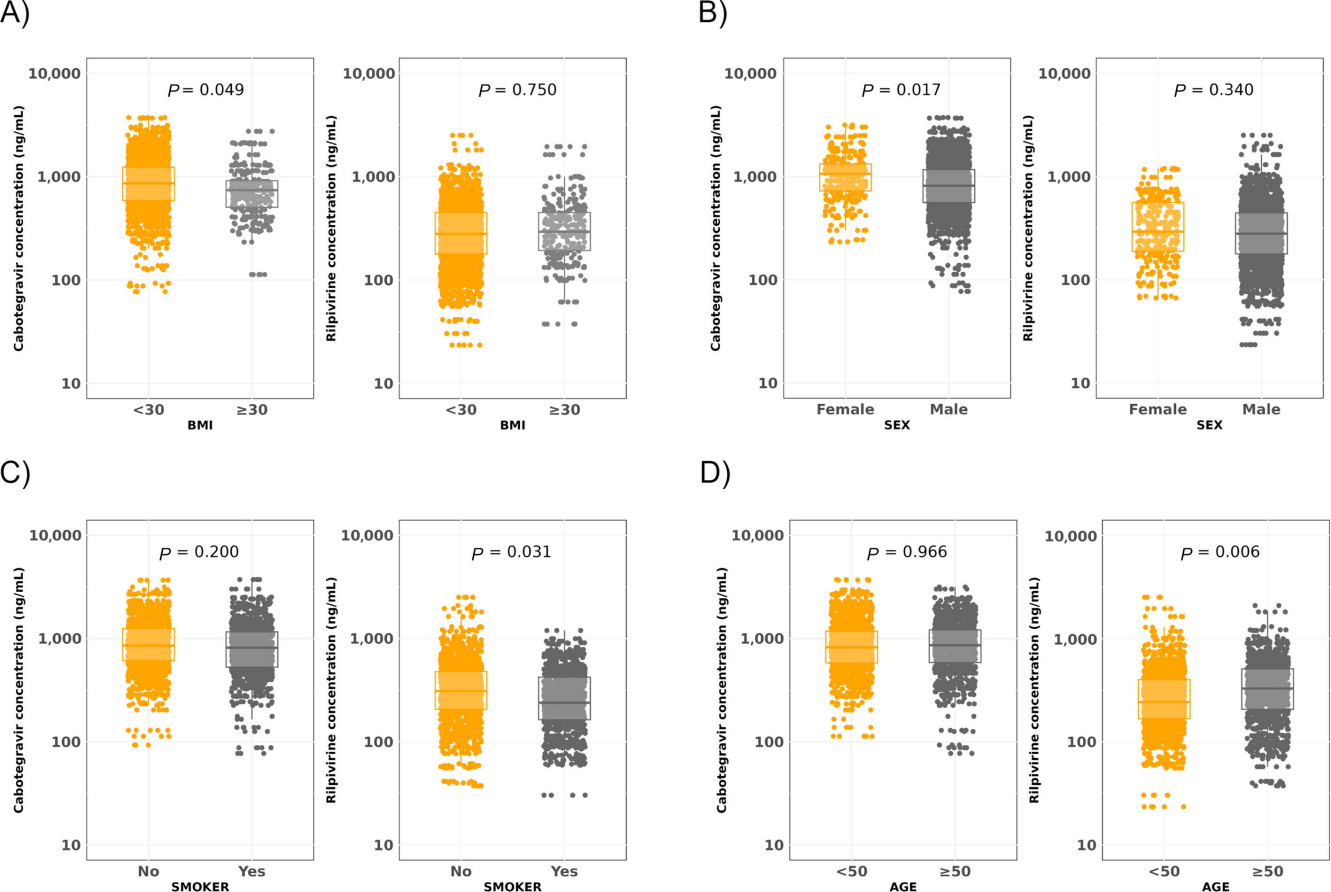
Distribution of all trough plasma concentrations of cabotegravir and rilpivirine determined in study participants according to (**A**) body mass index (BMI) in Kg/m^2^, (**B**) sex, (**C**) smoking habit, and (**D**) age in years. Linear mixed-effects models were used to assess the impact of individual patient-specific factors on CAB and RPV trough levels.

Univariate analysis of trough concentrations throughout the follow-up revealed that lower median CAB trough levels were significantly associated with BMI ≥30 kg/m² (2.87 [2.70–2.96] vs. 2.93 [2.76–3.09] log_10_ ng/mL, *P* = 0.049) and male sex (2.90 [2.74–3.07] vs. 3.02 [2.85–3.12], *P* = 0.017). Lower RPV trough concentrations were linked to smoking (2.39 [2.22–2.65] vs. 2.49 [2.32–2.69], *P* = 0.031) and younger age (<50 years) (2.40 [2.23–2.60] vs. 2.54 [2.35–2.71], *P* = 0.006) (Fig. 1). CAB levels were inversely correlated with BMI during the initiation phase (months 1 and 3) and with male sex during the maintenance phase (months 3–7) (Fig. S3).

**Fig 2:**
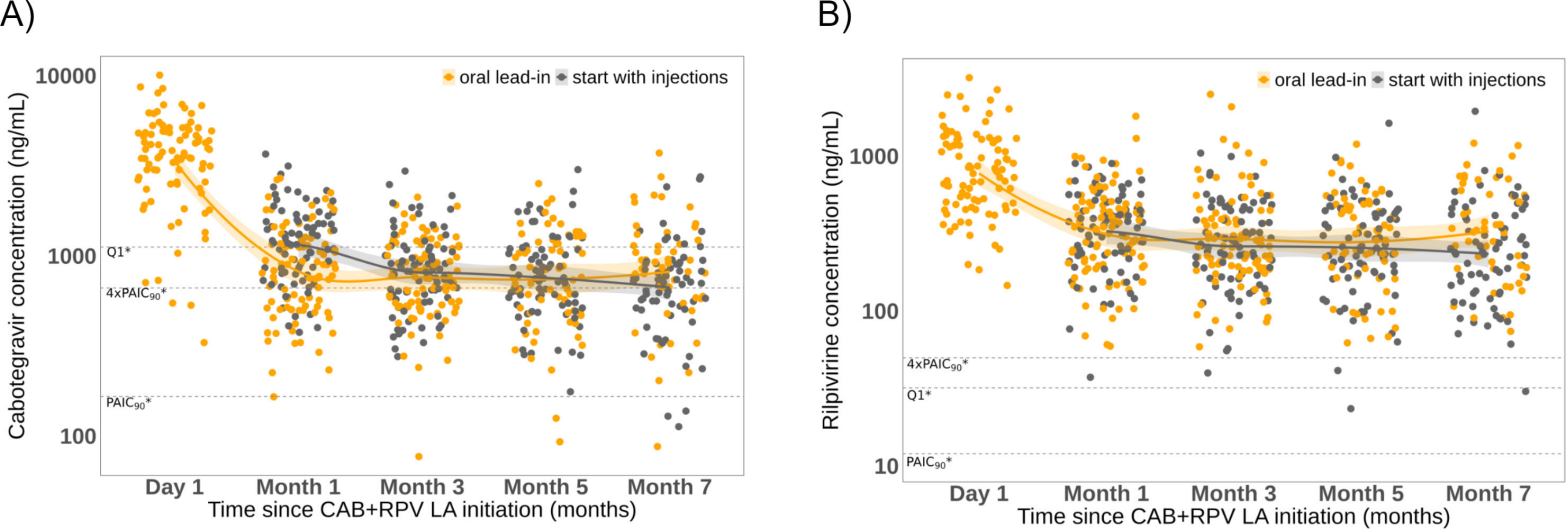
(**A**) Cabotegravir and (**B**) rilpivirine trough plasma concentrations over the 7-month follow-up period in patients starting with an oral lead-in or directly with injections. Trend lines fitted to trough concentrations of each group using local regression (LOESS) (95% CI). CAB, cabotegravir; LA, long-acting; RPV, rilpivirine. *Reported thresholds from clinical trials: *in vitro* protein-adjusted inhibitory concentration required for 90% viral inhibition (PAIC90): 166 ng/mL for CAB, 12 ng/mL for RPV; 4xPAIC90: 664 ng/mL for CAB and 50 ng/mL for RPV; and Q1 Ctrough, 25th percentile: 1,120 ng/mL for CAB and 32 ng/mL for RPV (references 1–4).

Overall, the median CAB and RPV trough levels were comparable between participants with and without an OLI phase (*P* = 0. 241 and *P* = 0.155, respectively). Throughout the study period, 99% (1,206/1,214) of concentrations exceeded the protein-adjusted inhibitory concentration (PAIC90) thresholds established in clinical trials (166 ng/mL for CAB and 12 ng/mL for RPV) (Fig. 2; Table S2). Potential drug-drug interactions (DDIs) with LA CAB + RPV identified in both groups are summarized in Table S3. No cases of co-medication interacting with CAB were identified during follow-up. However, three potential DDIs affecting RPV concentrations were observed: (i) sporadic use of clarithromycin in the SWI group, (ii) sporadic use of fluconazole in the OLI group, and (iii) a woman in the OLI group who received six cycles of ABVD chemotherapy. Despite this, all of them maintained good pharmacokinetic control and sustained virological suppression.

### Predictors of lower cabotegravir and rilpivirine levels

**Fig 3:**
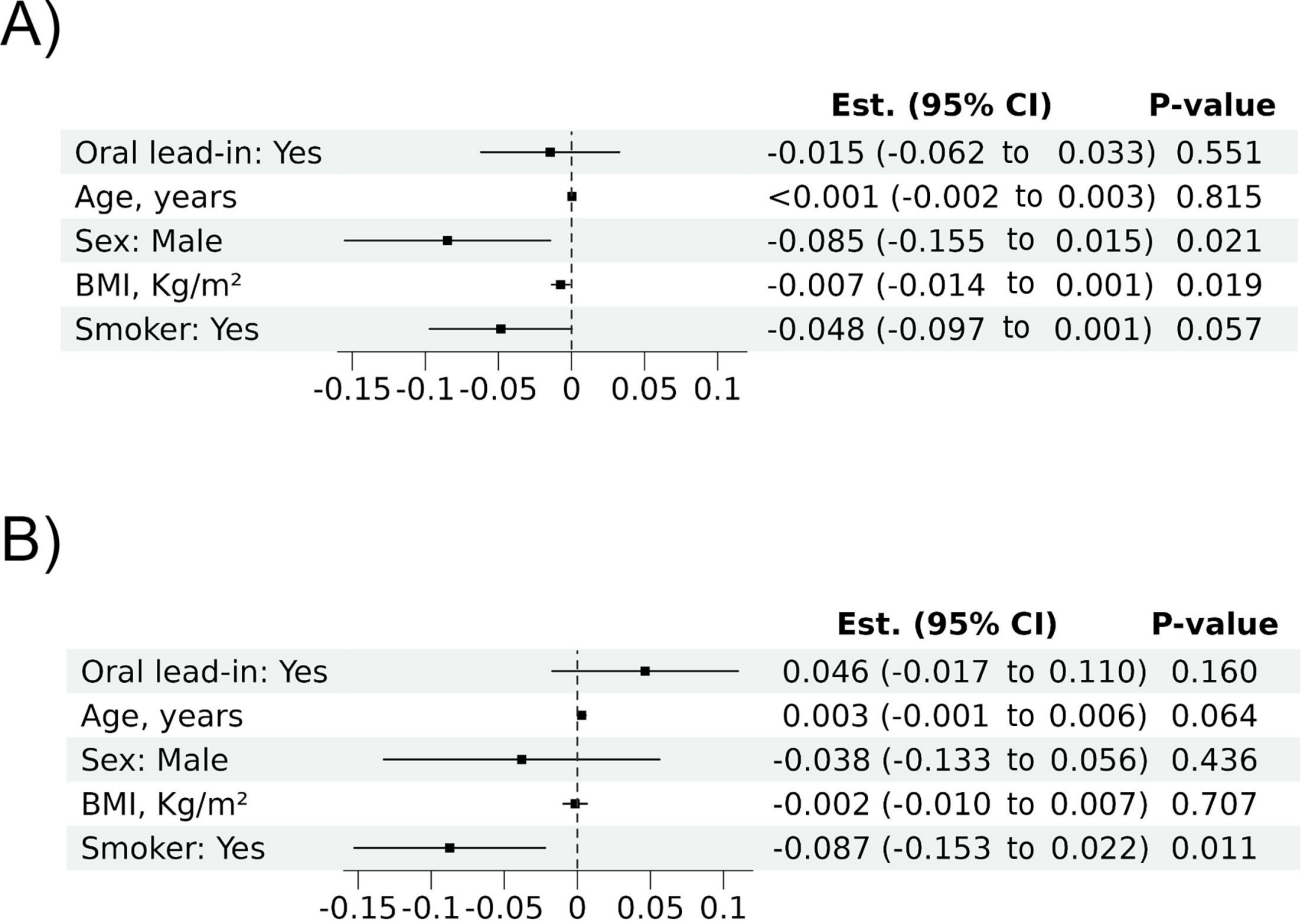
Linear mixed-effects models of baseline predictors of (**A**) cabotegravir and (**B**) rilpivirine trough plasma concentrations over the 7-month follow-up period. BMI, body mass index (kg/m^2^); Est, regression model adjusted-estimates; CI, confidence interval.

Predictive modeling using an adjusted linear mixed-effects model identified patient-specific factors as significant predictors of CAB and RPV plasma concentrations. For CAB, higher BMI (*P* = 0.019), male sex (*P* = 0.021), and smoking (*P* = 0.057) were associated with reduced trough plasma levels. For RPV, smoking (*P* = 0.011) and younger age (*P* = 0.064) were significant predictors of lower concentrations. Notably, the use of an OLI phase did not significantly affect CAB or RPV trough plasma levels (Fig. 3; Table S4). Secondary analyses of participants with baseline VL <50 copies/mL (*N* = 165) showed similar trends (Table S5).

## DISCUSSION

This study underscores the significant influence of patient-specific factors—particularly BMI, sex, and smoking status—on the PK of LA CAB + RPV therapy in PWH. These findings demonstrate that individual characteristics play a more significant role in drug exposure than the use of an OLI phase. Importantly, our results confirm that omitting the OLI phase does not compromise pharmacological efficacy or virological suppression, supporting a more patient-centered and flexible approach to initiating LA CAB + RPV therapy.

Our analysis revealed no significant differences in CAB or RPV trough concentrations or virological outcomes between participants who transitioned directly to injections and those who underwent a 4-week OLI phase. Both groups consistently maintained plasma drug levels above PAIC90 thresholds, achieving comparable rates of virological suppression and low rates of virological failure. These findings align with prior research, including the FLAIR and SOLAR trials, which demonstrated similar efficacy and PK profiles between SWI and OLI initiation strategies (4, 5, 10, 13).

Although earlier studies, such as Rubenstein *et al*. (6), reported lower CAB concentrations in SWI participants during the initial treatment phase, our larger cohort, extended follow-up period, and comprehensive PK measurements revealed comparable drug exposure between groups by the mid-term follow-up. Consistent with Rubenstein *et al*. (6), virological failure rates in the SWI group were not elevated, reinforcing the conclusion that effective viral suppression can be achieved without an OLI phase. By including a diverse real-world cohort and extending the observation period, our study strengthens the evidence supporting simplified therapy initiation to enhance accessibility and reduce barriers to PWH.

In addition to the initiation strategy, our findings highlight the impact of patient-specific factors on CAB and RPV pharmacokinetics. A higher BMI was significantly associated with reduced CAB concentrations, particularly during the early treatment phase. This corroborates previous reports that increased adipose tissue can influence drug distribution and metabolism (9). However, the impact of BMI diminished over time, consistent with findings by Cossu et al. (14), suggesting that although BMI may affect initial drug exposure, it does not impair long-term therapeutic efficacy.

Sex-based differences also emerged, with male participants demonstrating lower CAB concentrations during the maintenance phase. This observation aligns with earlier studies (7–9) and may reflect physiological differences, including variations in body composition and enzyme activity that influence drug metabolism and distribution. Furthermore, smoking status was a significant predictor of reduced CAB and RPV concentrations, supporting evidence that smoking may induce hepatic UGT1A1 (15) and CYP3A4 (16) enzymes, which are involved in CAB and RPV metabolism, respectively, albeit to varying degrees. This induction could potentially accelerate drug metabolism and clearance (9, 17). These findings emphasize the importance of accounting for individual physiological and lifestyle factors when prescribing LA therapy, as they may substantially influence drug exposure and clinical outcomes. Additionally, careful consideration of DDIs is essential, as these interactions may affect the effectiveness and safety of LA therapy or concomitant medications, potentially leading to adverse events, viral rebound, or the development of ART drug resistance.

The omission of the OLI phase offers several practical advantages. It simplifies treatment initiation, reduces logistical challenges, and facilitates faster access to LA therapy—particularly in resource-limited settings where managing additional clinical visits can be difficult. Although the OLI phase may still be valuable in specific cases, such as for patients with known tolerability concerns or unique PK profiles, our results suggest it should not be universally required.

This study has certain limitations. Although the single-center design ensures methodological consistency, it may restrict the generalizability of the findings to broader and more diverse populations. For plasma concentration measurements, unlike previous studies (6–8, 10), we employed lyophilized deuterated internal standards with commercial RPV and CAB suspensions, as well as thermal inactivation in compliance with institutional safety procedures (18). These methodological differences limit the cross-sectional comparability of our plasma levels with prior research. Finally, the observational nature of this study introduces the potential for unmeasured confounders, such as genetic polymorphisms (19) that may influence drug metabolism, which were not accounted for in the analysis.

Future research should explore the interplay between genetic variations, BMI, sex, and smoking on CAB and RPV pharmacokinetics. Such investigations could inform precision dosing strategies and further personalize LA therapy for PWH, optimizing outcomes for diverse patient populations.

In conclusion, this study demonstrates that patient-specific factors—BMI, sex, and smoking—are more influential determinants of CAB and RPV pharmacokinetics than the use of an OLI phase. These findings support flexible and individualized initiation strategies for CAB + RPV LA therapy, prioritizing patient-tailored care to enhance treatment accessibility and effectiveness while ensuring optimal outcomes for diverse populations.

## Data Availability

The data that support this work are available from the corresponding author upon reasonable request.

## SUPPLEMENTAL MATERIAL

Supplemental material aac.00145-25-s0001.docx Tables S1 to S5; Fig. S1 to S3.

ASM does not own the copyrights to Supplemental Material that may be linked to, or accessed through, an article. The authors have granted ASM a non-exclusive, world-wide license to publish the Supplemental Material files. Please contact the corresponding author directly for reuse.
