# Supplementary material for: Influence of patient characteristics and oral lead-in on long-acting cabotegravir and rilpivirine pharmacokinetics and outcomes in people with HIV: a real-world study
Source: Antimicrob Agents Chemother. 2025 Jun 5;69(7):e00145-25. doi: 10.1128/aac.00145-25 (PMC12217485; doi:10.1128/aac.00145-25)

**SUPPLEMENTAL MATERIAL**

**Influence of Patient Characteristics and Oral Lead-In on Long-Acting Cabotegravir and Rilpivirine Pharmacokinetics and Outcomes in People with HIV: A Real-World Study**

**Table of contents**

Supplementary TABLE S1…………….……………………………………………………2

Supplementary TABLE S2…………….……………………………………………………3

Supplementary TABLE S3…………….……………………………………………………4

Supplementary TABLE S4…………….…………………………………………………….6

Supplementary TABLE S5…………….…………………………………………………….6

Supplementary FIGURE S1…………………………………………………………………7

Supplementary FIGURE S2………………………………………...………………………8

Supplementary FIGURE S3………………………………………….…………….……….9

**TABLE S1.** Characteristics of the three participants with virological failure during the study period.

| **Characteristic** | **Virological Failure Cases** | | |
| --- | --- | --- | --- |
| Age in years (y), gender | 48 y, female | 45 y, male | 49 y, male |
| HIV subtype | B | B | B |
| RPV-associated mutations in historical medical records | NNRTI-associated mutation: 103N | No | No |
| BMI, Kg/m2 | 23.7 | 31.3 | 31.0 |
| Pre-transition ART regimen | DTG/RPV | BIC/FTC/TAF | DTG/LMV |
| CAB+RPV Oral lead-in | No | Yes | Yes |
| Time point on CAB+RPV at VF | Month 9 | Month 1 | Month 3 |
| Plasma viral load at VF, copies/mL (time point at which the sample was obtained) | 928 (Month 9)  285 (Month 11) | 2780 (Month 1)  4260 (Month 3) | 45600 (Month 3)  15400 (Month 5)  51 (Month 6)  461 (Month 7) |
| INSTI-associated mutations at VF and drug resistance interpretation^a^ | 145PS, 148R | 148Q/R, 263K | No |
| CAB  DTG  BIC  EVG  RAL | High-Level  Low-Level  Low-Level  High-Level  High-Level | High-Level  Intermediate  Intermediate  High-Level  High-Level |  |
| NNRTI-associated mutations at VF and drug resistance interpretation^a^ | 100LI, 103N, 181yC | 138K | 181C |
| DOR  EFV  ETR  NVP  RPV | Intermediate  High-Level  High-Level  High-Level  High-Level | Susceptible  Low-Level  Low-Level  Intermediate  Intermediate | Susceptible  Intermediate  Intermediate  High-Level  Susceptible |
| CAB trough plasma levels, ng/mL (time point at which the sample was obtained) | 592 (Month 1)  666 (Month 3)  301 (Month 5)  725 (Month 7)  629 (Month 9) | 2507 (Oral lead-in)  1535 (Month 1)  773 (Month 3) | 330 (Oral lead-in)  278 (Month 3)  301 (Month 5) |
| RPV trough plasma levels, ng/mL (time point at which the sample was obtained) | 192 (Month 1)  243 (Month 3)  196 (Motnh 5)  199 (Month 7)  439 (Month 9) | 849 (Oral lead-in)  633 (Month 1)  293 (Month 3) | 416 (Oral lead-in)  110 (Month 3)  99 (Month 5) |
| Post-VF-ART regimen (time point at which the regimen was started) | DTG/FTC/TAF  (Month 11)^b^ | DRV/COB/FTC/TAF  (Month 4) | DRV/COB/FTC/TAF  (Month 7)  DTG/DRV/COB/FTC/TAF  (Month 12) |
| Post-VF plasma viral load, copies/mL (time point at which the sample was obtained) | 96 (Month 12)  <20 (Month 17) | 94 (Month 5)  22 (Month 9)  49 (Month 14) | 246 (Month 11)  142 (Month 12)  155 (Month 16) |

Abbreviations: ART, antiretroviral therapy; BMI, body mass index (calculated as weight in kilograms divided by height in meters squared); BIC, bictegravir; CAB, cabotegravir; COB, cobicistat; DOR, doravirine; DRV, darunavir; DTG, dolutegravir; EFV, efavirenz; EVG, elvitegravir; ETR, etravirine; FTC, emtricitabine; INSTI, integrase strand transfer inhibitor; LA, long acting; LMV, lamivudine; NVP, nevirapine; NNRTI, non-nucleoside reverse transcriptase inhibitor; RAL, raltegravir; RPV, rilpivirine; TAF, tenofovir alafenamide; VF, virological failure. **^a^** According to the Stanford University HIV Drug Resistance Database (<https://hivdb.stanford.edu/>). ^b^ The participant started with DRV/COB/FTC/TAF in month 11 but switched to DTG/FTC/TAF the next day due to intolerance.

**TABLE S2.** Cabotegravir and rilpivirine trough plasma concentrations during the study visits according to initiation strategy.

|  | **Cabotegravir Ctrough** | | |  | **Rilpivirine Ctrough** | | |
| --- | --- | --- | --- | --- | --- | --- | --- |
|  | **Oral lead-in** | **Start with injections** | **P** |  | **Oral lead-in** | **Start with injections** | **P** |
| **All** | N=298 | N=309 |  | **All** | N=298 | N=309 |  |
| median(IQR), ng/mL | 825 (528-1170) | 823 (607-1217) | 0.241 | median(IQR), ng/mL | 296 (193-484) | 280 (175-445) | 0.155 |
| median(IQR), log10 ng/mL | 2.92 (2.72-3.07) | 2.92 (2.78-3.09) |  | median(IQR), log10 ng/mL | 2.47 (2.29-2.68) | 2.45 (2.24-2.65) |  |
| <166 ng/mL (PAIC_90_)*, N (%) | 5 (1.7) | 3 (1.0) | 0.834 | <12 ng/mL (PAIC_90_)*, N (%) | 0 | 0 | - |
| <664 ng/mL (4xPAIC_90_)*, N (%) | 112 (37.6) | 101 (32.7) | 0.222 | <50 ng/mL (4xPAIC_90_)*, N (%) | 0 | 5 (1.6) | 0.643 |
| <1120 ng/mL (Q1)*, N (%) | 208 (69.8) | 209 (70.9) | 0.841 | <32 ng/mL (Q1)*, N (%) | 0 | 2 (0.7) | 0.499 |
| **Month 1** | N=90 | N=80 |  | **Month 1** | N=90 | N=80 |  |
| median(IQR), ng/mL | 874 (520-1184) | 1178 (838-1561) | <0.001 | median(IQR), ng/mL | 344 (198-484) | 368 (237-471) | 0.674 |
| **Month 3** | N=91 | N=79 |  | **Month 3** | N=91 | N=79 |  |
| median(IQR), ng/mL | 773 (542-1150) | 812 (599-1089) | 0.703 | median(IQR), ng/mL | 277 (195-394) | 271 (168-450) | 0.280 |
| **Month 5** | N=65 | N=77 |  | **Month 5** | N=65 | N=77 |  |
| median(IQR), ng/mL | 761 (492-1194) | 737 (546-1083) | 0.845 | median(IQR), ng/mL | 286 (189-490) | 260 (179-430) | 0.470 |
| **Month 7** | N=52 | N=73 |  | **Month 7** | N=52 | N=73 |  |
| median(IQR), ng/mL | 856 (586-1177) | 738 (542-903) | 0.035 | median(IQR), ng/mL | 338 (191-618) | 242 (135-414) | 0.013 |

Abbreviations: Ctrough, trough concentrations; IQR, interquartile range; PAIC_90_, in vitro protein-adjusted inhibitory concentration required for 90% viral inhibition; Q1, 25th percentile. *CAB and RPV trough plasma concentrations thresholds as reported in the literature (references 1-4).

**TABLE S3.** Cases of Drug-Drug Interactions with Long-Acting CAB+RPV.

| **Patient (Age, Sex)** | **LA Group** | **Active Ingredients** | **Route of Administration** | **Usage^a^** | **Indication** | **Long-acting CAB+RPV Drug-Drug Interactions (DDIs)^a^** | | |
| --- | --- | --- | --- | --- | --- | --- | --- | --- |
|  |  |  |  |  |  | **LA CAB+RPV** | **CAB** | **RPV** |
| 67 y, Male | SWI | Diazepam/  Sulpiride | Oral | Habitual | Anxiety | No/  Potential | No | QT prolongation risk |
| 49 y, Male | SWI | Mirtazapine | Oral | Habitual | Anxiety | Potential Weak | No | Possible risk of QTc prolongation and/or TdP |
| 53 y, Male | SWI | Escitalopram | Oral | Habitual | Anxiety/depression | Potential | No | QT prolongation risk |
| 61 y, Female | SWI | Levofloxacin | Oral | Punctual (7 days) | Respiratory infection | Potential | No | QT prolongation risk |
|  |  | Clarithromycin | Oral | Punctual (7 days) |  | Potential | No | Expected increase of RPV exposure (inhibition of CYP3A enzymes). QT prolongation risk |
| 53 y, Male | SWI | Methadone | Oral | Habitual | Opioid dependence | Potential | No | Decreased methadone concentrations, clinical monitoring is recommended. QT prolongation risk |
|  |  | Budesonide/  Formoterol | Inhaled | Habitual | COPD exarcebation | No/Potential Weak | No | Possible risk of QTc prolongation |
| 59 y, Female | OLI | Escitalopram | Oral | Habitual | Anxiety | Potential | No | QT prolongation risk |
| 64 y, Male | OLI | Venlafaxine | Oral | Habitual | Depression | Potential Weak | No | Possible risk of QTc prolongation and/or TdP |
| 51 y, Male | OLI | Amiodarone | Oral | Habitual | Paroxysmal tachycardia | Potential | No | QT prolongation risk |
| 51 y, Male | OLI | Azithromycin | Oral | Punctual (3 days) | Tonsillitis | Potential | No | QT prolongation risk |
| 35 y, Male | OLI | Fluconazole | Oral | Punctual (1 day) | Balanitis | Potential | No | Possible increase in RPV plasma levels (inhibition of CYP3A enzymes). QT prolongation risk |
| 60 y, Female^c^ | OLI | Ondansetron | Oral | 30 days | Chemotherapy (antiemetic) | Potential | No | QT prolongation risk |
|  |  | Bleomycin | IV | 3 cycles | Hodking lymphoma (ABVD chemotherapy) | No | No | No |
|  |  | Doxorubicin | IV | 6 cycles |  | Potential | No | Possible cardiac toxicities (ECG abnormalities and sometimes arrhythmias). QT prolongation risk |
|  |  | Vinblastine | IV |  |  | Contraindicated | No | Vinblastine is metabolized by CYP3A4 and significantly decrease RPV concentrations, potentially leading to loss of therapeutic effect and the development of resistance |
|  |  | Dacarbazine | IV |  |  | No | No | No |
| 42 y, Male | OLI | Levofloxacin | Oral | Punctual (7 days) | Pneumonia | Potential | No | QT prolongation risk |
| 25 y, Male | OLI | Azithromycin | Oral | Punctual (3 days) | Nasopharyngitis | Potential | No | QT prolongation risk |
| 63 y, Male | OLI | Methadone | Oral | Habitual | Opioid dependence | Potential | No | Decreased methadone concentrations, clinical monitoring recommended. QT prolongation risk |
| 31 y, Male | OLI | Azithromycin | Oral | Punctual (5 days) | Influenza symptoms | Potential | No | QT prolongation risk |
| 57 y, Male | OLI | Mirtazapine | Oral | Habitual | Anxiety-opioid dependence | Potential Weak | No | Possible risk of QTc prolongation and/or TdP |
| 56 y, Male | OLI | Mirtazapine | Oral | Habitual | Depression | Potential Weak | No | Possible risk of QTc prolongation and/or TdP |

ABVD, chemotherapy combination (doxorubicin, bleomycin, vinblastine, dacarbazine); CAB, cabotegravir; IV, intravenous; OLI, oral lead-in; RPV, rilpivirine; SWI, start with injections; TdP, Torsade de Pointes. ^a^ Usage frequency during the 11-month LA follow-up. ^b^ Drug-drug interactions (DDIs) identified using the University of Liverpool HIV Drug Interaction Database, categorized as “potential weak interaction”, “potential interaction“ and “contraindicated”. ^c^ Participant undergoing chemotherapy; regular HIV-1 viral load monitoring showed sustained viral suppression during and after chemotherapy.

**TABLE S4.** Linear mixed-effects models of baseline predictors of cabotegravir and rilpivirine trough plasma concentrations over the 7-month follow-up.

|  | **Cabotegravir** (log_10_ ng/mL) | | **Rilpivirine** (log_10_ ng/mL) | |
| --- | --- | --- | --- | --- |
|  | Estimates (95% CI) [p value] | | | |
|  | Un-adjusted or  Crude | Adjusted (N=166) ^a^ | Un-adjusted | Adjusted (N=166) ^a^ |
| Age, years | <0.001 (-0.002—0.003) [.615] | <0.001 (-0.002—0.003) [.815] | 0.003 (-0.001—0.006) [.056] | 0.003 (-0.001—0.006) [.064] |
| Sex, male | **-0.086 (-0.156— -0.017) [.016]** | **-0.085 (-0.155— -0.015) [.021]** | -0.048 (-0.143—0.047) [.321] | -0.038 (-0.133—0.056) [.436] |
| BMI, Kg/m^2^ | **-0.007 (-0.013— -0.001) [.025]** | **-0.007 (-0.014— -0.001) [.019]** | 0.001 (-0.007—0.009) [.830] | -0.002 (-0.010—0.007) [.707] |
| Smoker | -0.032 (-0.080—0.017) [.203] | -0.048 (-0.097—0.001) [.057] | **-0.066 (-0.129— -0.002) [.044]** | **-0.087 (-0.153— -0.022) [.011]** |
| OLI use | -0.021 (-0.068—0.026) [.390] | -0.015 (-0.062—0.033) [.551] | 0.038 (-0.026—0.101) [.249] | 0.046 (-0.017—0.110) [.160] |

Abbreviations: CI, confidence interval; OLI, oral lead-in; ^a^ Models conducted using data from 166 participants with complete records. Bolded values represent statistically significant predictors (*P* < 0.05). All models included a random intercept for patients.

**TABLE S5.** Linear mixed-effects models of baseline predictors of cabotegravir and rilpivirine trough plasma concentrations over the 7-month follow-up for participants with VL <50 copies/mL at baseline.

|  | **Cabotegravir** (log_10_ ng/mL) | **Rilpivirine** (log_10_ ng/mL) |
| --- | --- | --- |
|  | Estimates (95% CI) [p value]  Adjusted (N=159) ^a^ | |
| Age, years | <0.001 (-0.002—0.003) [.779] | 0.003 (-0.001—0.006) [.075] |
| Sex, male | **-0.100 (-0.173— -0.025) [.010]** | -0.064 (-0.164—0.037) [.221] |
| BMI, Kg/m^2^ | **-0.008 (-0.014— -0.002) [.014]** | -0.002 (-0.010—0.007) [.692] |
| Smoker | **-0.053 (-0.103— -0.002) [.044]** | **-0.090 (-0.158— -0.022) [.011]** |
| OLI use | -0.024 (-0.072—0.025) [.347] | 0.038 (-0.027—0.104) [.262] |

Abbreviations: CI, confidence interval; OLI, oral lead-in; ^a^ Models conducted using data from 159 participants with complete records. Bolded values represent statistically significant predictors (*P* < 0.05). All models included a random intercept for patients.

**Figure S1.** HIV-1 RNA levels in plasma during the study visits according to initiation strategy. Percentages of plasma samples with HIV-1 RNA <50 copies/mL. P >0.05 for the comparison between groups. Baseline, day of first intramuscular LA injection. Month 1, 3, 5, 7, 9 and 11: one, three, five, seven, nine and eleven months after the first intramuscular injection, respectively. *Percentage of patients with all plasma samples with HIV-1 RNA <50 copies/mL over the 11-month follow-up. Median (IQ) number of VL ≥50 copies/mL per participant with low-level HIV-1 viremia was 1(1-2).


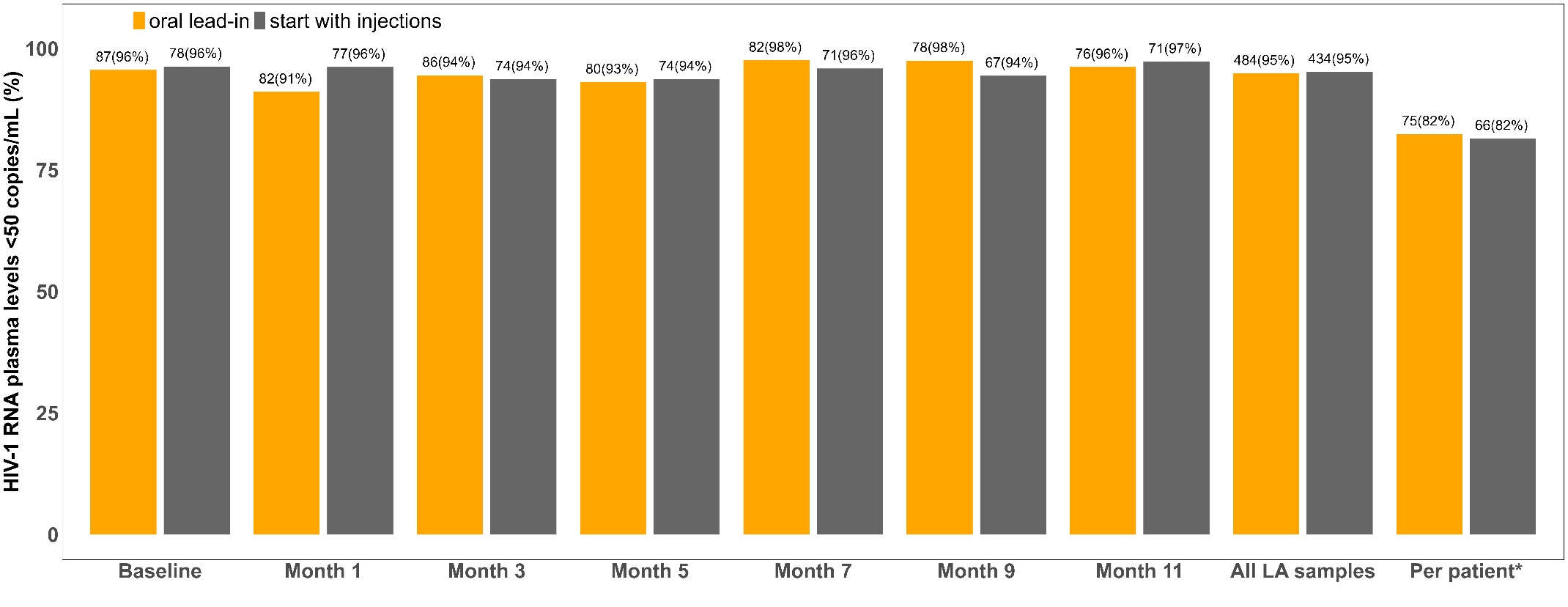


**Figure S2.** Distribution of A) cabotegravir and B) rilpivirine trough plasma concentrations determined in study participants. CAB, cabotegravir; LA, long-acting; PAIC_90_, in vitro protein-adjusted inhibitory concentration required for 90% viral inhibition; RPV, rilpivirine.


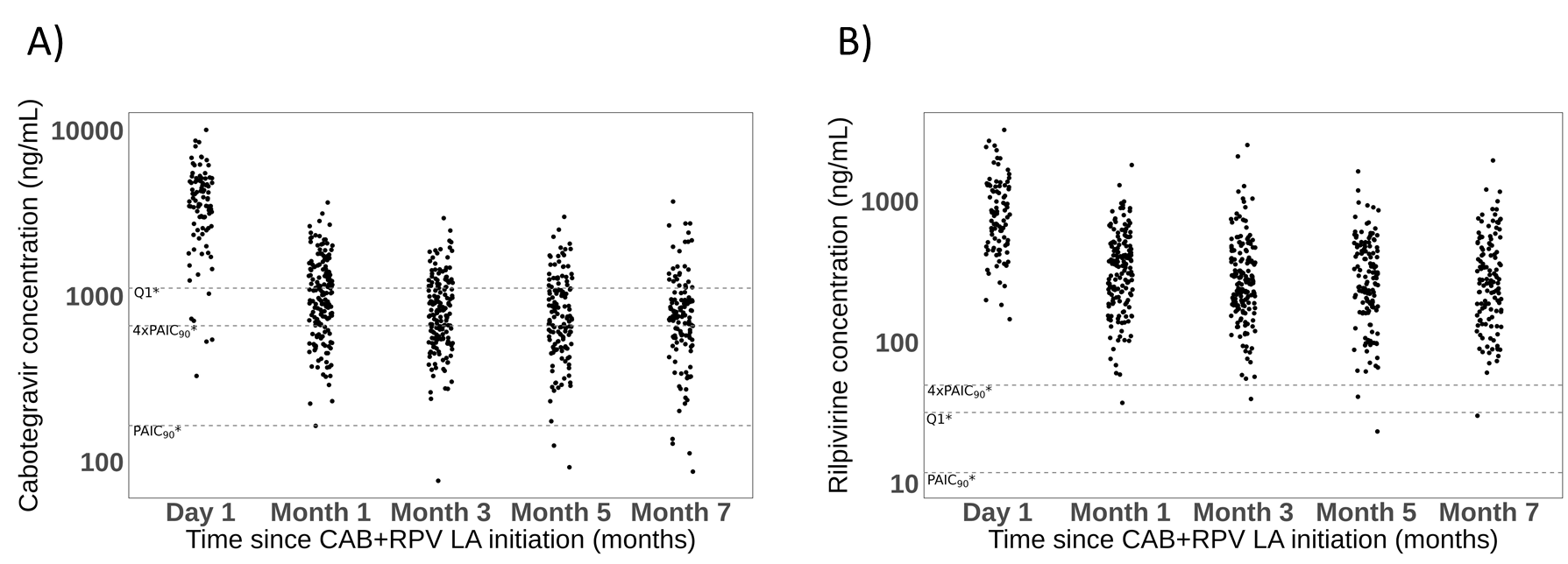


**Figure S3.** Cabotegravir trough plasma concentrations according to A) sex and B) BMI (kg/m^2^) over the 7-month follow-up period. Trend-lines fitted to trough concentrations of each group using local regression (LOESS) (95% confidence interval). BMI, body mass index; CAB, cabotegravir; RPV, rilpivirine.


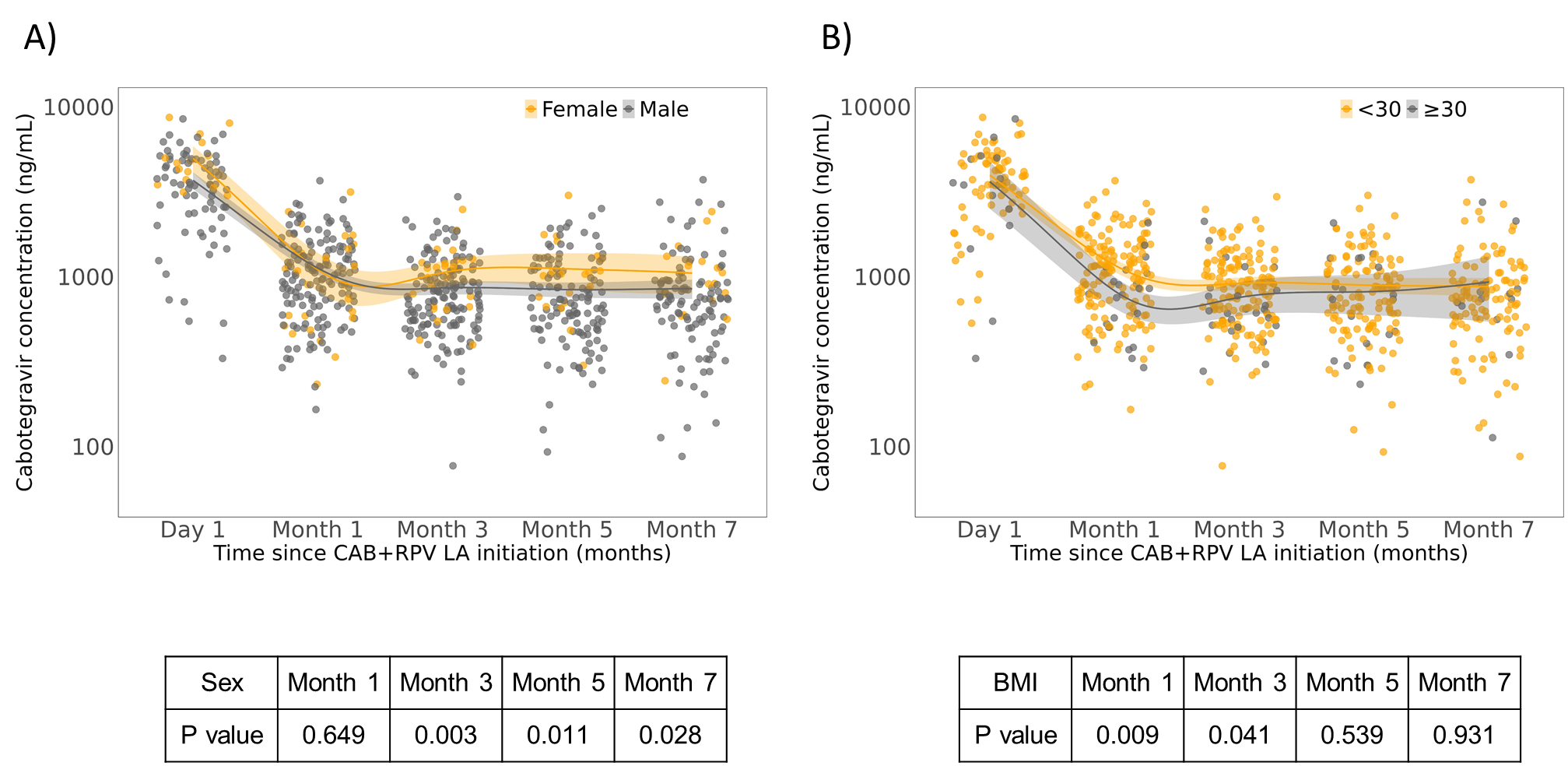

Supplement: Supplemental material — Tables S1 to S5; Fig. S1 to S3. [file aac.00145-25-s0001.docx]
